# How to Treat Chronic Idiopathic Testicular Pain? Scrotoscopy with a Novel Percutaneous Endoscopy Equipment

**DOI:** 10.1155/2018/9808152

**Published:** 2018-09-20

**Authors:** Junhao Lei, Chunhua Luo, Xinjun Su, Xinghuan Wang

**Affiliations:** ^1^Department of Urology, Zhongnan Hospital of Wuhan University, Wuhan University, Wuhan 430071, China; ^2^Operating Room, Department of Urology, Zhongnan Hospital of Wuhan University, Wuhan University, Wuhan 430071, China; ^3^Center for Evidence-Based and Translational Medicine, Wuhan University, Wuhan 430071, China

## Abstract

**Background:**

Management of chronic idiopathic testicular pain may be difficult because of problems identifying the causes. We evaluated “AUTOKLAV”, a novel endourological nephrolithotomy device to diagnose and treat chronic idiopathic testicular pain.

**Methods:**

We divided 103 patients to either scrotoscopy group (SG, n = 64) or open exploration group (OEG, n = 39) between September 2014 and March 2017 at Zhongnan Hospital. Perioperative information, like operating time, length of incision, and wound infections, was carefully recorded during in hospital. Follow-up data, like pain scores improvement, satisfaction with penis appearance, and adverse event, were collected at one month postoperatively.

**Results:**

Finally, both the operating time and length of incision showed better performance for SG (43.6 ± 4.7 versus 51.5 ± 9.0 min; 0.7 ± 0.2 versus 4.1 ± 0.8 cm; both P <0.01). Though the pain improvement had no significant differences between the two groups (2.92 ± 0.99 and 2.14 ± 1.02, p>0.05), SG showed obvious advantages in incidence of wound infections and satisfaction with wound/scrotum appearance (0% versus 2.9%; 96.4% versus 85.3%, both P<0.05).

**Conclusions:**

In conclusion, scrotoscopy using the novel AUTOKLAV device is feasible, has an acceptable complication rate, and can be effective and safe in men with idiopathic chronic testicular pain. Etiologically, secondary inflammatory changes caused by the complete or incomplete torsion of testicular or epididymis appendices or by the existence of stones in the tunica sac might be responsible for the pain.

## 1. Introduction

Patients with chronic testicular pain who present with intermittent or constant unilateral or bilateral testicular pain persisting for >3 months are frequently encountered by urologists or general practitioners. This condition considerably interferes with the patient's daily activities [1]. It can be disabling, and many patients seek medical attention because of anxiety. The management of chronic testicular pain is often difficult and time consuming, especially for those without identified causes. Considering the lack of effective treatment methods, patients have to rely on long-term analgesic medication, which often does not provide remarkable relief. The goal of treatment is to facilitate patient return to routine activity and promote patients' quality of life; meanwhile, adverse events (AEs) caused by the treatment should be little and slight.

Chronic testicular pain can have well-defined testicular causes or even be idiopathic [2]. Unfortunately, the cause of chronic idiopathic testicular pain cannot be determined through a routine examination in majority of patients [3]. Conventional open exploration always leads to many AEs, such as a long incision in the scrotum, hematoma, and serious edema, which prolong recovery. The introduction of scrotoscopy may have brought new hope to these patients. Scrotoscopy was first described by Shafik et al. [4], and its safety was preliminarily verified by Franco et al. in the 1990s [5]. It is a novel endourologic technique that explores the scrotum and easily provides a direct and clear view of the testis and epididymis. Data on the clinical applications of scrotoscopy for managing patients with scrotal diseases are limited. Franco et al. evaluated 20 cadavers and only five patients; hence, they could not provide sufficient evidence pertaining to the safety and efficacy of this procedure. In 2014, Yan et al. [6] were the first to treat testicular hydrocele with the aid of a scrotoscope, providing a viable and encouraging technique for the diagnosis and treatment of hydrocele. Furthermore, in 2016, Ye et al. [7] verified that scrotoscopy was a minimally invasive, safe, and effective approach for the early diagnosis of testicular torsion.

To the best of our knowledge, no study has reported on the use of a scrotoscope to manage chronic idiopathic testicular pain. In this paper, we compared the safety and clinical efficacy between conventional open exploration and novel scrotoscopy using an endourological nephrolithotomy device named AUTOKLAV. In addition, the possible causes of chronic idiopathic testicular pain were explored.

## 2. Materials and Methods

### 2.1. Clinical Data

This trial was undertaken at Zhongnan Hospital, Wuhan University, Wuhan, China. Between September 2014 and February 2017, 103 patients aged between 19 and 65 years were enrolled. The patients had no history of surgery for scrotal contents and had chronic testicular pain persisting for >3 months. Patients with testicular pain owing to testicular causes, such as tumors, torsion, and trauma, were excluded after routine physical examination, ultrasound, or other diagnostic procedures. Patients with varicocele, inguinal hernia, intervertebral disc prolapse, or urinary stone disease were also excluded. Patients were given the freedom to choose between the two procedures: open exploration or scrotoscopy. However, the patients were sufficiently informed of the merits of the two methods before deciding on which one to choose. The study was performed in accordance with the ethical principles of the Declaration of Helsinki. All patients gave informed consent prior to being included. The study was approved by the Ethics Committee of Zhongnan Hospital of Wuhan University. The registration ID of this clinical trial at Chinese Clinical Trial Registry was ChiCTR-INR-17012652.

### 2.2. Surgical Instruments and Method

**Figure 1:**
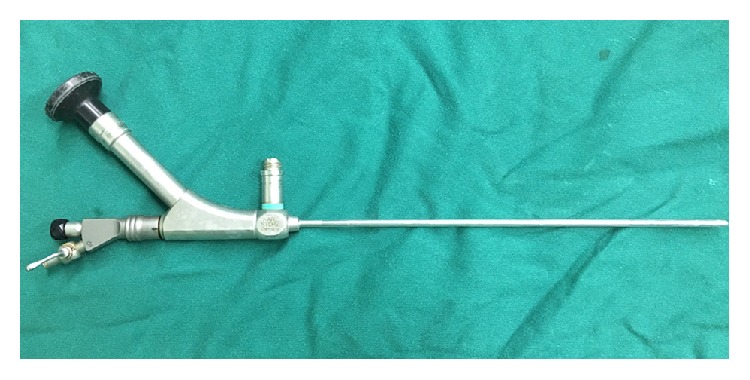
The instruments of scrotoscope named AUTOKLAV (KARL STORZ, Germany). Actually, it was a kind of mini-percutaneous nephrolithotomy equipment, which was designed for endoscopic treatment of kidney stone, with endoscope as well as working and irrigating channel.

In the scrotoscopy group (SG), AUTOKLAV (KARL STORZ, Germany) was used for scrotoscopy because no specialized devices were available (Figure 1) [8–10]. This was a novel percutaneous mini-nephrolithotomy instrument set that was designed for the endoscopic treatment of kidney stones. It comprised an endoscope as well as working and irrigation channels. A medical holmium laser (Ho:YAG) therapeutic apparatus (Wuxi Dahua Laser Equipment Co., LTD, Wuxi, China) was used to break scrotal stones or vaporize testicular or epididymal appendices.

**Figure 2:**
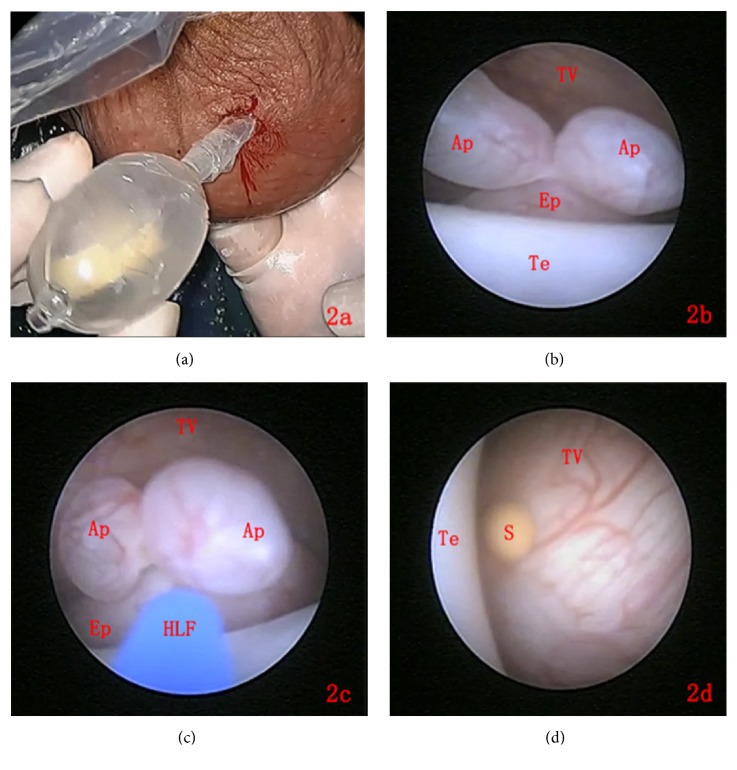
The key processes of scrotoscope procedures using AUTOKLAV. 2(a) The homemade working sheath was passed through the incision into tunica vaginalis space, allowing AUTOKLAV inserting into the scrotum from its tail. 2(b) The epididymal appendices were detected with an appearance like two fingers. 2(c) The base of appendices was vaporized by the holmium laser fiber. 2(d) A stone was detected near the tail of the epididymis with an appearance like pearl.** TV**: tunica vaginalis;** Ap**: appendices;** Ep**: epididymis;** Te**: testis;** HLF**: holmium laser fiber;** S**: stone.

The procedure was performed under sterile conditions, with the patient in a lithotomy position. Spinal anesthesia was administered because (1) it has a lower adverse reaction rate and (2) the patients could resume food intake and return to daily activity early (6 h after surgery). A minimal incision (5–10 mm) was made using a scalpel blade at the anterior inferior part of the lesion side of the scrotum, and layer-by-layer dissection was performed until entry into the tunica sac. After three Allis clamps were used to hold all the layers of the scrotal wall, a homemade working sheath was passed through the incision into the tunica sac and then steadily fixed to the scrotal wall using 2-0 sutures (Figure 2(a)), allowing the insertion of the scrotoscope (through the inner channel) into the tunica sac. The tunica sac was distended using 40–60 mL of isotonic crystalloid solution, with continuous irrigation through the inner channel of the scrotoscope. The fluid was kept freely flowing out of the scrotum through the space between the sheath and scrotoscope to maintain a balance between the irrigation fluid volume and pressure in the scrotum throughout the procedure. The testis, epididymis, and tunica vaginalis were carefully examined via the scope under a clear field of direct vision. Operating forceps or laser fiber could be used throughout the procedure under visualization with the scope. During the surgery, when testicular or epididymal appendices were detected (Figure 2(b)), the appendices were drawn out through the working channel using biopsy forceps after vaporizing the base of the appendices using the holmium laser fibers (18–30 W) (Figure 2(c)). Scrotal stones could also be broken using the holmium laser (Figure 2(d)). After the scrotoscope and sheath were displaced, the artificial hydrocele was drained by compression of the scrotum, followed by suturing of the incision.

In the open exploration group (OEG), the surgical position and anesthesia method were similar to those in SG. A 3–5-cm incision was made in the scrotal surface, and the scrotal wall was opened till the tunica sac. The testis and epididymis were removed through the incision, and all the contents were exposed and directly examined. All abnormalities were identified and treated before the incision was sutured layer by layer.

After surgery, scrotal bandages were well fixed in both groups, and the first dressing change was performed within 24 h. The subsequent dressing changes were performed according to the extent of recovery of the wound. Surgical procedures in both groups were performed by a single surgeon (SXJ).

### 2.3. Observational Indexes and Follow-Ups

Perioperative information, including duration of pain, operating time, length of incision, blood loss, incidences of scrotal hematoma and edema, number of testis and/or epididymis injuries, number of wound infections, and hospitalization days, was recorded during the procedure and before discharge. Duration of pain was defined as the duration from the beginning of pain to the recruitment time. Operating time was defined as the duration from the incision in the scrotum to the termination of suturing. Intraoperative blood loss was calculated based on the increase in weight of the standard gauze. The visible, suspected causes of chronic testicular pain were also recorded during the procedure.

Patients were requested to visit our department for follow-up at 1 month after the surgery. Physical examination was performed with special attention to the external genitalia. A visual analog scale (VAS) [8] was used to evaluate the pain on a scale of 0–10, with 0 representing no pain and 10 representing sharp pain. Pain relief was calculated by recording the pain scores at two time points: before treatment and at 1 month after the surgery. The blood flow to the testis and epididymis was evaluated using B-mode ultrasound. Other AEs such as wound infections, hematoma, and edema were also well evaluated by a single surgeon (SXJ) to minimize the error. Satisfaction with wound and scrotal appearance was also recorded.

### 2.4. Statistical Analysis

All the statistical analyses were performed using a statistical software package (SPSS, Version 16.0, Chicago, IL, USA). Continuous variables are expressed as mean ± standard deviation (SD). Discrete variables are expressed as percentages. T test was used to compare continuous data, and* χ*^*2*^ tests were used for discrete data. Statistical significance was defined as a P value of <0.05. All P values were two-sided. Two authors (LJH and GYM) conducted the statistical analysis independently, and any disagreements were resolved through discussion among the study group members.

## 3. Results

**Figure 3:**
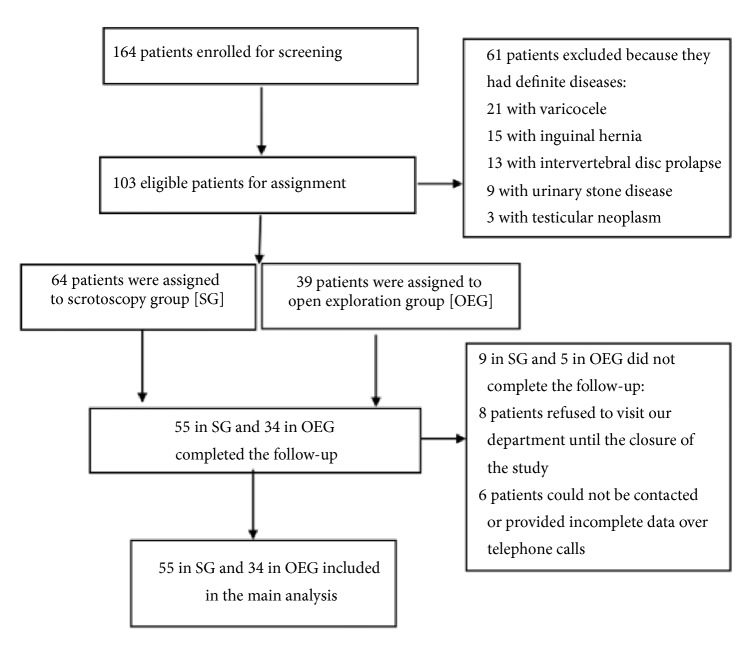
The TREND flowchart of the patients through each stage of the study.

The TREND flowchart of the patients through each stage of the study was shown at Figure 3. Finally, a total of 103 patients were assigned to the following two groups: SG (n = 64) and OEG (n = 39). Between the two groups, there were no significant differences in the mean age, average pain score, and duration of pain (mean age: 42.3 ± 8.4 versus 41.6 ± 6.9 years, P = 0.06; pain score: 4.75 ± 1.07 versus 4.07 ± 1.16, P = 0.32; duration of pain: 8.3 ± 2.7 versus 7.7 ± 1.9 min, P = 0.09).

All procedures were performed successfully in the 103 patients. The operating time in SG was much less than those in OEG (43.6 ± 4.7 versus 51.5 ± 9.0 min, P < 0.01). In SG, based on intraoperative observation, the blood loss was very little but could not be easily evaluated because of the continuous irrigation with isotonic crystalloid solution (not available versus 15.7 g, respectively). The length of the incision in SG was significantly shorter than that in OEG (0.7 ± 0.2 versus 4.1 ± 0.8 cm; P < 0.01). The incidence of scrotal edema (at postoperative day 2; 3.1% versus 7.7%, P < 0.05) and hematoma (during hospitalization; 0% versus 5.1%) was less in SG than in OEG. In addition, SG had a shorter duration of hospitalization (4.7 ± 0.9 versus 7.1 ± 0.8 days; P < 0.01). Both types of surgeries did not result in any testis or epididymis injuries. Perioperative comparisons between the two groups are given in Table 1.

**Figure 4:**
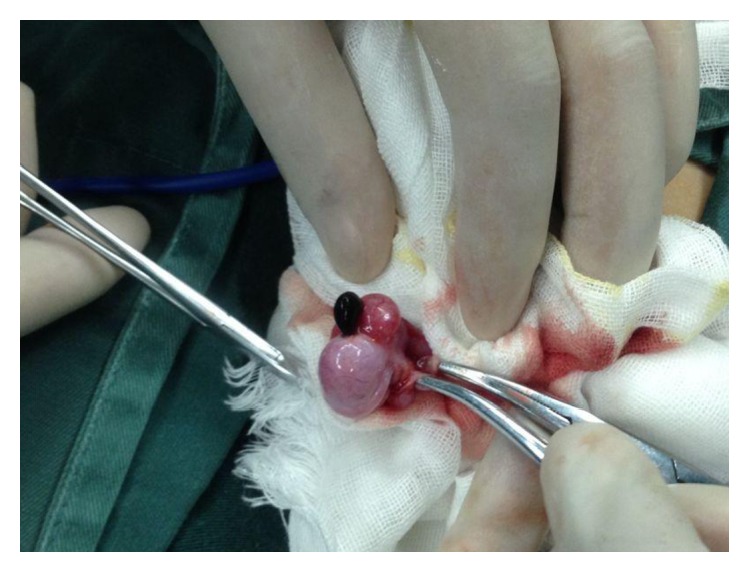
Appendices necrosis due to torsion. This picture exhibited a case in the open exploration group with appendices necrosis showing black colour because of torsion.

**Figure 5:**
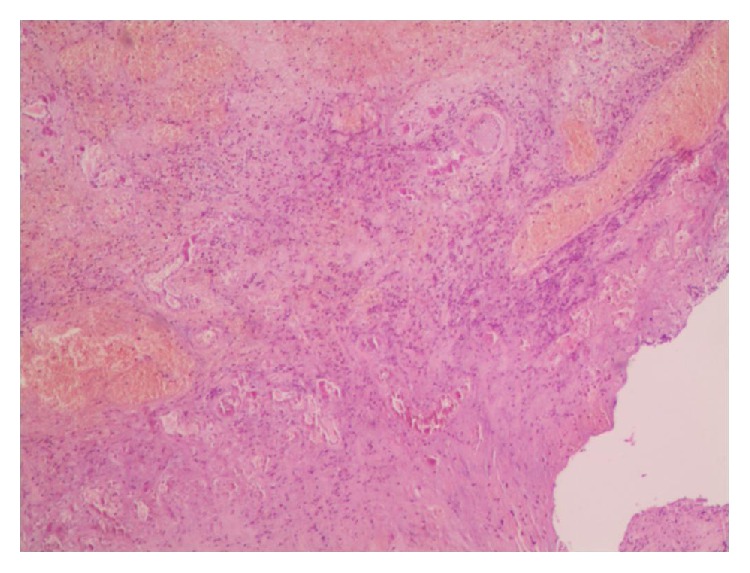
HE staining slice of epididymal appendices. The ranges of vision are filled with simple columnar epithelium or simple cuboidal epithelium. The fibrous connective tissues are degenerative. Original magnification ×40.

During the procedure, testicular or epididymis appendices were detected in all 103 cases, and scrotal stones were found in 32 cases (24 in SG and 8 in OEG).  Figure 4 shows an OEG case wherein the appendices were necrosed because of torsion. Pathological examination revealed a simple cyst lined with simple columnar epithelium or simple cuboidal epithelium in all appendices, with different degrees of inflammatory cell infiltration for most patients (94/103) (Figure 5).

At the 1-month follow-up, complete data of 89 patients (55 in SG and 34 in OEG) were available, indicating a follow-up rate of 86.4%. Of the remaining 14 patients who were lost to follow-up, 8 patients refused to visit our department for further consultation until the closure of the study and 6 patients could not be contacted or provided incomplete data over telephone calls. At 1 month after the procedures, in the 89 patients with successful follow-up, the mean pain scores in SG and OEG were 1.82 ± 0.82 and 1.93 ± 0.92, respectively (P > 0.05). The improvement in pain scores in SG and OEG was 2.92 ± 0.99 and 2.14 ± 1.02, respectively (P > 0.05), which did not significantly differ between the groups. On the other hand, the pain scores before and after surgery were significantly different, both for SG (P < 0.05) and for OEG (P < 0.05). The blood flow was normal, and no testicular/epididymal ischemia or necrosis was detected by B ultrasound. In addition, no significant differences were noted with respect to the occurrence of hematoma (0% versus 0%) or edema (0% versus 0%). Nevertheless, patients in SG showed obvious advantages over those in OEG in terms of the incidence of wound infections and satisfaction with wound/scrotum appearance (0% versus 2.9% and 96.4% versus 85.3%, respectively; P < 0.05). The follow-up data at one month postoperatively are shown in Table 2.

## 4. Discussion

Chronic testicular pain has multiple causes, such as testicular tumor, torsion, varicocele, and trauma, and management depends on the definite etiology of testicular pain. Unfortunately, despite using ultrasound, computed tomography, and magnetic resonance imaging, the etiology of chronic idiopathic testicular pain is not easily determined. Open exploratory operation can serve as an alternative, but the acceptability of this procedure is low among patients because of its invasiveness. Patients urgently require new methods to determine the etiology and treat their pain with procedures that are safe, effective, and minimally invasive.

As described by Shafik et al. [4] and Franco et al. [5], scrotoscopy has been reported to be minimally invasive and allows for a direct view to evaluate the scrotal contents and harvest tissue samples for evaluating the pathophysiology. In China, Guang et al. [9] used scrotoscopy in 1990 to perform a biopsy of the testes of infertile men, and Yang et al. [10] used scrotoscopy in 1996 to diagnose scrotal lesions. According to Yang's data, scrotoscopy was far more precise than B ultrasound in distinguishing a benign lump from a tumor because the former allowed biopsy and removal of the lump under direct vision.

Recently, novel reports on the clinical application and progress of scrotoscopy have earned praise for innovation and commendable efforts. In 2014, Yan et al. [6] conducted minimal hydrocelectomy using a scrotoscope. They used a resectoscope as a scrotoscope to examine the intrascrotal contents and eliminate any pathological lesions, following which the parietal tunica vaginalis was excised through a 2-cm scrotal incision. No major complications occurred during the postoperative follow-up period. Thereafter, Wang et al. [11] published their experience in the diagnosis and management of scrotal superficial angiomyxoma using a scrotoscope. Their results demonstrated that a scrotoscope is a good tool for locating the mass in the scrotum and ensuring its complete removal, thereby decreasing the recurrence rate. In addition, Yang et al. [12] randomized 48 patients with symptomatic epididymal cysts (ECs) to receive either traditional open epididymal cystectomy (OEC, n = 23) or minimal epididymal cystectomy with resectoscope as a scrotoscope (MECS, n = 25). Significant differences were observed after MECS compared with after OEC based on the rates of symptomatic relief (95.2% versus 61.1%; P < 0.05) and duration of wound pain (12.1 versus 17.7 days; P < 0.05). Their study proved that MECS may be a safe, effective, and encouraging technique for EC treatment. In 2016, Ye et al. [7] employed a pediatric cystoscope as a scrotoscope to examine the testis/epididymis of patients with acute onset of scrotal pain and verified that it was a minimally invasive, safe, and effective approach for the early diagnosis of testicular torsion (100% specificity and sensitivity).

Unfortunately, no studies have reported on the management of chronic testicular pain using scrotoscopy, and we presume that the limited application of scrotoscopy may result from an inappropriate use of instruments and the limited number of available cases. To the best of our knowledge, this is the first prospective trial to compare the effectiveness and safety between scrotoscopy and open exploration for the management of chronic idiopathic testicular pain. We used an AUTOKLAV (Figure 1), which is a novel percutaneous mini-nephrolithotomy instrument set that provides clear visualization with irrigation and a working channel for treatment. Its outer diameter is only 2 mm; hence, the length of the incision in the scrotum is minimized. The length of the incision was significantly shorter in SG than in OEG (0.7 ± 0.2 versus 4.1 ± 0.8 cm; P < 0.01), thereby providing patients with a satisfactory wound/scrotum appearance (96.4% versus 85.3%, P < 0.05).

Regarding the primary endpoint, the improvement in pain scores at 1 month was higher in SG than in OEG, although without significant differences (2.92 ± 0.99 versus 2.14 ± 1.02, P > 0.05). Even so, either method, scrotoscopy or open exploration, could significantly relieve their pain (P < 0.05). The higher pain improvement might be related to the less impact of scrotoscopy on the scrotal contents due to the use of auxiliary tools, such as biopsy forceps and fiber optic lasers, for biopsy, excision, and vaporization throughout the examination and treatment procedures. Conversely, open exploration might more easily lead to damage, scars, or adhesions when exposing the testicle, epididymis, and spermatic cord outside the scrotum; furthermore, compared with using scrotoscopy, the anatomical structures of the scrotal contents were more severely damaged using this procedure. This finding can also explain why the incidences of scrotal edema (3.1% versus 7.7%, P < 0.05) and hematoma (0% versus 5.1%) were significantly less in SG than in OEG. A comparison of our SG method with Yang's scrotoscopy method [12] revealed that the latter used a resection electrode loop, which led to a higher incidence of edema (2/25, 8%). Conversely, we used operating forceps or laser fibers, which theoretically had less impact on the scrotal wall and contents. In addition to the above-mentioned reason, scrotal edema in SG was partly caused by the extravasation of saline solution into the subdartoic space and gradually resolved spontaneously over 3 days after the procedure.

Regarding the etiology, our results are encouraging with respect to an indication of scrotoscopy for chronic testicular pain, particularly in cases where no definite cause is determined on routine examination. The findings of the 103 cases were finally confirmed by pathologic examination. Cystic masses with simple columnar or simple cuboidal epithelium were observed, with different degrees of inflammatory cell infiltration for most patients (94/103). They were considered to be testicular or epididymal appendices (Figure 5). In addition, scrotal stones were detected in 32 cases (24 in SG and 8 in OEG). After the removal of the appendices and stones, both the groups exhibited satisfactory pain improvement (SG versus OEG: 2.92 ± 0.99 versus 2.14 ± 1.02). Therefore, it is reasonable to assume that the existence of appendices and stones was responsible for the chronic idiopathic testicular pain. In the tunica sac, the dynamic friction between the torsioned appendices/stones and tunica vaginalis would lead to secondary inflammatory changes in the scrotal wall and contents, exacerbating the testicular pain. Particularly, in the case described in Figure 4, the patient suffered from increasing testicular pain in the first few days and then with intermittent pain. This condition was finally confirmed to be caused by a complete torsion of the testicular appendages. This hypothesis was also verified by Karmazyn et al. [13], who reported that in 29 patients diagnosed with torsion of the testicular appendages based on testicular exploration, secondary inflammatory changes, including hydrocele in 22 (75.9%), enlarged epididymis in 22 (75.9%), scrotal wall edema in 16 (55.2%), and swollen testis in 9 (31%) patients, commonly occurred.

Like other studies, this study also has limitations. Given the rare nature of the disease, we were only able to include <100 eligible participants despite a 2.5-year recruitment period. Another limitation is that this was a nonrandomized study. Data obtained from multicenter randomized studies with longer follow-up period can further validate the clinical effects of this surgical procedure in men with chronic idiopathic testicular pain.

In conclusion, scrotoscopy using the novel AUTOKLAV device is feasible, has an acceptable complication rate, and can be effective and safe in men with idiopathic chronic testicular pain. Secondary inflammatory changes caused by the complete or incomplete torsion of testicular or epididymis appendices or by the existence of stones in the tunica sac might be responsible for the chronic idiopathic testicular pain.

## Figures and Tables

**Table 1:** The baseline data and perioperative outcomes when comparing “scrotoscopy group” with “open exploration group”.

Items	SG (N=64)	OEG (N=39)	P value (2-sided/tailed)
**Baseline data**			
Age (years)	42.3±8.4	41.6±6.9	0.06
Duration of pain (months)	8.3±2.7	7.7±1.9	0.09
Pain scores for preoperation	4.75±1.07	4.07±1.16	0.32
**Perioperative data**			
Operation time (min)	43.6±4.7	51.5±9.0	<0.01
Blood loss	NA	15.7	NA
Length of incision (cm)	0.7±0.2	4.1±0.8	<0.01
Scrotal edema, n (%)			
*2 days postoperation*	2 (3.1%)	3(7.7%)	<0.05
Scrotal hematoma, n (%)			
*during in hospital*	0 (0%)	2 (5.1%)	NA
Testis /epididymis injury, n (%)	0 (0%)	0 (0%)	NA
Hospitalization duration (d)	4.7±0.9	7.1±0.8	<0.01

SG: scrotoscopy group. OEG: open exploration group. NA: not available.

**Table 2:** The follow-up data at one month postoperatively.

Items	SG (N=55)	OEG (N=34)	P value (2-sided/tailed)
Follow-up rate	85.9%	87.2%	>0.05
Pain scores^&^			
*One mon postoperation*	1.82±0.82	1.93±0.92	>0.05
*Scores improvement*^∧^	2.92±0.99	2.14±1.02	>0.05
Scrotal edema, n (%)			
*One mon postoperation*	0 (0%)	0 (0%)	NA
Scrotal hematoma, n (%)			
*1 mon postoperation*	0 (0%)	0 (0%)	NA
Testicular/epididymis necrosis	0 (0%)	0 (0%)	NA
Wound infections, n (%)	0 (0%)	1 (2.9%)	NA
Satisfaction with wound /scrotum appearance, n (%)	53(96.4%)	29(85.3%)	<0.05

SG: scrotoscopy group. OEG: open exploration group. ^&^The pain scores before and after surgery were significantly different, both for SG (P < 0.05) and for OEG (P < 0.05). ^∧^The improvement in pain scores between SG and OEG showed no significant difference. NA: not available.

## Data Availability

The data used to support the findings of this study are available from the corresponding author upon request.

## Supplementary Materials

Supplementary Materials Along with the manuscript, we also uploaded a short video named “Video 1.mp4” as supplementary material file to show the treatment process via the scrotoscopy.
